# Broadband Transient Response and Wavelength-Tunable Photoacoustics in Plasmonic Hetero-nanoparticles

**DOI:** 10.1021/acs.nanolett.3c00063

**Published:** 2023-03-16

**Authors:** Anton Yu. Bykov, Yuanyang Xie, Alexey V. Krasavin, Anatoly V. Zayats

**Affiliations:** Department of Physics and London Centre for Nanotechnology, King’s College London, London WS2R 2LS, U.K.

**Keywords:** Plasmonics, opto-acoustics, nanoparticles, optical nonlinearities

## Abstract

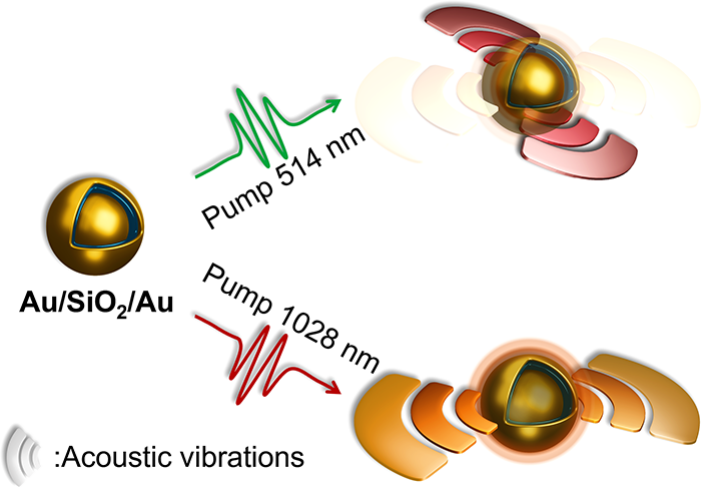

The optically driven acoustic modes and nonlinear response of plasmonic nanoparticles are important in many applications, but are strongly resonant, which restricts their excitation to predefined wavelengths. Here, we demonstrate that multilayered spherical plasmonic hetero-nanoparticles, formed by alternating layers of gold and silica, provide a platform for a broadband nonlinear optical response from visible to near-infrared wavelengths. They also act as a tunable optomechanical system with mechanically decoupled layers in which different acoustic modes can be selectively switched on/off by tuning the excitation wavelength. These observations not only expand the knowledge about the internal structure of composite plasmonic nanoparticles but also allow for an additional degree of freedom for controlling their nonlinear optical and mechanical properties.

Plasmonic nanostructures exhibiting strong electromagnetic field enhancement in the spectral vicinity of localized surface plasmon (LSP) resonances have been an active topic of research leading to many exciting applications in the fields of nanoscale sensing,^1a^ optoelectronics,^1^ data storage, and biomedical applications,^1b^ to name but a few. For smooth metal films, the hot carrier excitation and related nonlinear and transient effects can be induced primarily under the optical excitation in the spectral range corresponding to the interband transitions where the efficient light absorption takes place or while exciting propagating surface plasmon polaritons.^2^ The strongest third-order Kerr-type nonlinear response and the associated transient changes are observed in the same spectral range. At the longer wavelength of the excitation and probing, in the visible and near-infrared spectral ranges, the nonlinear response is orders of magnitude smaller.^3^ Plasmonic nanoparticles supporting LSPs, provide both the enhanced absorption and the resonant scattering near the plasmonic resonance, so that the nonlinearity can be excited and observed in the relatively narrow spectral range near the LSP. Alternatively, a metamaterial approach can be used to design a spectrum of the nonlinearity enhancement at the epsilon-near-zero regime.^4^

Complex plasmonic systems combining several elements either in a metamaterial framework^5^ or in free space^6,7^ offer additional degrees of freedom to control optical resonances with respect to individual plasmonic nanoparticles. One example is the multilayered metal–dielectric nanoparticles (metaparticles) formed by alternating metal and dielectric layers.^7−15^ Such nanoparticles have been used in refractive index sensing, enhanced Raman scattering and fluorescence, nonlinear optics, and biomedical applications.^10−12,16^ The optical spectrum of these nanoparticles, which increases in complexity with the number of metal–dielectric layers can be understood as a result of hybridization between plasmon modes of multiple plasmonic shells, allowing designer optical properties to be achieved.^7,17^

Acoustic vibrations of nano-objects have attracted significant attention due to the ease of their excitation by optical means, the potential to study mechanical phenomena at nanometer length scales, and the key role they play in the thermal dissipation processes and coupling to vibrational modes of molecules, which in turn may influence molecular reactions.^18−21^ Thus, the methods to control and selectively excite acoustic vibrations are important for the investigation of fundamental physical and chemical properties and processes. Pump–probe measurements were successfully used to study lattice phonons in thin films and plasmonic nanoparticles^22,23^ and the interaction of acoustic waves with nanostructures.^24^ The interplay between optically excited acoustic and plasmonic modes in a plasmonic crystal has been used to achieve a coherent control of the plasmonic modes.^25^

Generally, acoustic vibrations are sensitive to the size, morphology, and composition of the objects.^26,27^ Structural design is one of the approaches for the underlying vibrational modes’ manipulation. Optical excitation of structurally anisotropic nano-objects, such as nanorods and nanocrosses, where multiple fundamental vibration modes exist because of lower symmetry of the structure, enables polarization-dependent control of the vibration modes due to the dependence of local polarizability on the polarization of light, resulting in a shift of the vibration frequency.^28,29^ For vibrating spherical bodies, possessing a ladder of mode overtones, the ability to redistribute the vibration energy between fundamental and high-order modes has been demonstrated with sequential excitation using several consequent optical pulses.^30^ This approach only allows control of the overtones of the same fundamental mode. Hetero-nanoparticles, such as multishell nanoparticles studied here, have potential to build an efficient platform for manipulating acoustic vibrations.

In this Letter, we show that multilayered Au/SiO_2_/Au hetero-nanoparticles possess unique broadband transient optical properties, including a rich spectrum of optically excited acoustic vibrations that can be selectively excited by tuning the excitation wavelength. The gold core and the gold shell can be excited to vibrate separately. The fundamental vibration mode of the gold core can only be observed when the excitation coincides with the particular mode in the absorption spectrum of the nanoparticles. In these nanoparticles with multiple plasmonic resonances, not only the broadband excitation of the transient response can be achieved scanning the spectrum of the available (overlapping) LSPs, but also the induced transient response, excited at one LSP resonance, can be probed in the vicinity of the other available LSP resonances, providing a broadband functionality.

Multilayered hetero-nanoparticles (Figure 1) were fabricated following a procedure described in ref (7) (see Methods for the details). The nanoparticle dimensions obtained from the TEM images are as follows: core diameter 57 ± 3 nm, SiO_2_ shell thickness 10 ± 2 nm, and a gold shell thickness 20 ± 6 nm.

**Figure 1:**
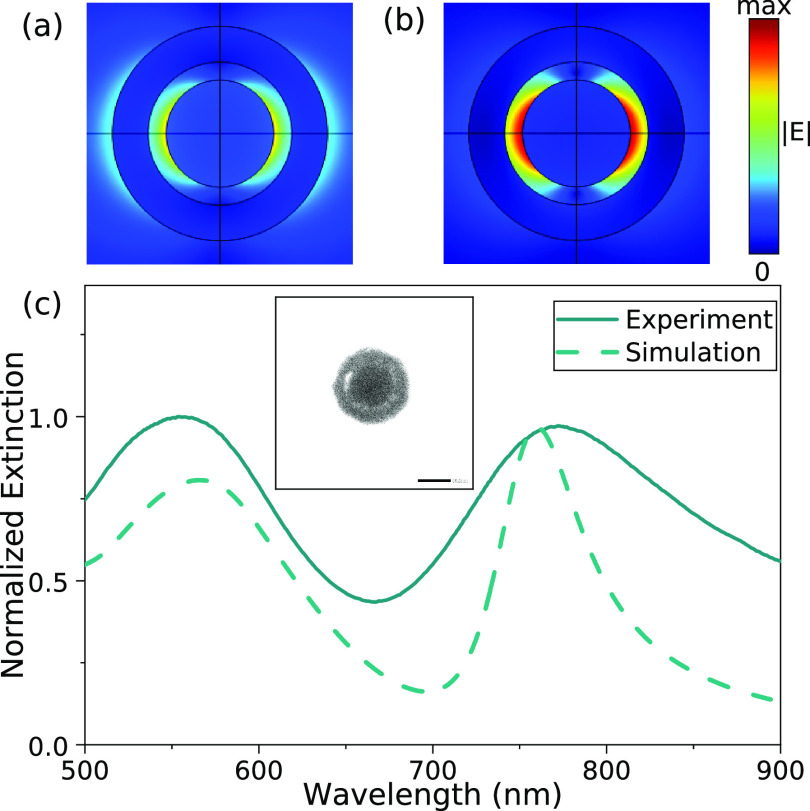
(a,b) Spatial distributions of the electric field amplitude corresponding to the extinction resonance wavelengths (a) 560 nm and (b) 760 nm. (c) Normalized extinction spectra of the Au/SiO_2_/Au nanoparticles. The inset shows the TEM image of a hetero-nanoparticle (the scale bar is 50 nm).

The experimental extinction spectra (Figure 1) reveal the double-resonant behavior consistent with the numerical simulations for the same parameters of the nanoparticles. The short-wavelength resonance around 560 nm can be identified as corresponding to the hybridized Au-nanoparticle/Au-nanoshell bonding mode mainly localized in the gold core.^7^ The resonance around 760 nm corresponds to the antibonding plasmon mode mainly localized in the gold shell. This resonance is wider in the experiment compared to the numerical simulations due to variation of the thicknesses of the gold shell that contributes to the inhomogeneous broadening.^31^

Transient absorption spectra of the nanoparticles were measured under the pulsed excitation at various wavelengths (see Supporting Information for the pump–probe experiment details), and, therefore, affecting different modes of the nanoparticles (Figure 2). Once the energy of the pump beam is absorbed in the nanoparticle (Figure 2b,d), the excitation of hot carriers in gold, followed by their thermalization, results in a complex transient spectrum with alternating regions of photoinduced absorption and transparency, consistent with shift and broadening of the two main extinction peaks.

**Figure 2:**
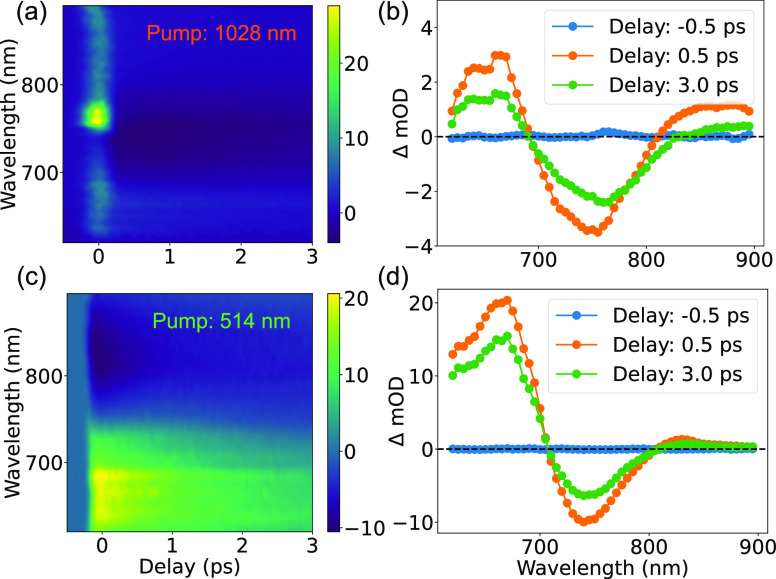
(a,c) Transient optical absorption spectra of the Au/SiO_2_/Au nanoparticles for (a) 1028 nm and (c) 514 nm excitation wavelengths. (b,d) Transient absorption spectra (cross sections of (a,c)) observed at the delays indicated in the panels.

The transient absorption spectra extend to a long-wavelength part of the spectrum (up to 900 nm in near-IR) making these multilayered nanoparticles a versatile tool for broadband optical manipulation and nonlinear response engineering. Compared to solid spherical gold nanoparticles for which only one absorption resonance is available, usually located around 550 nm, and the transient absorption signal is usually very weak in the near-IR,^32,33^ the hetero-nanoparticles benefit from availability of several, in some cases overlapping, spectral features.^7^ They can be addressed separately by adjusting the excitation wavelength.

No significant variations were observed in the decay times of the transient signal for multiple probe wavelengths and both excitation conditions, indicating that the same process of hot carrier cooling with emission of phonons is responsible for the temporal response. While photons with wavelength of 514 nm (2.4 eV) have enough energy to excite interband absorption around *L* and *X* high-symmetry points in the Brillouin zone of gold,^34^ the dynamics of hot holes in the d-band does not seem to contribute substantially to the observed decay.

Besides the transient absorption signal discussed above, a second, very-short-lived feature was observed for 1028 nm excitation, visible at close to zero delays (Figure 2a). This signal, also observed for pure deionized water without the nanoparticles, can be attributed to the coherent degenerate four-wave-mixing (FWM) process in the aqueous medium, and its spectrum is indicative of the vibrational levels of water molecules in the IR (see the Supporting Information).

The control over the excitation wavelength allows tuning the ratio of energy absorption in the gold core and the gold shell (Figure 3a,c). For the excitation at a wavelength of 514 nm, the energy density absorbed in the gold core is about 2 times larger than the energy density absorbed in the shell. The situation reverses for the 1028 nm excitation, when about 5 times more energy density is absorbed in the shell than in the core. The more energy is absorbed, the higher is the electron temperature rise in one or the other part of the hetero-nanoparticle. Therefore, two different excitation conditions introduce different distributions of the elevated electron temperature inside the nanoparticle.

**Figure 3:**
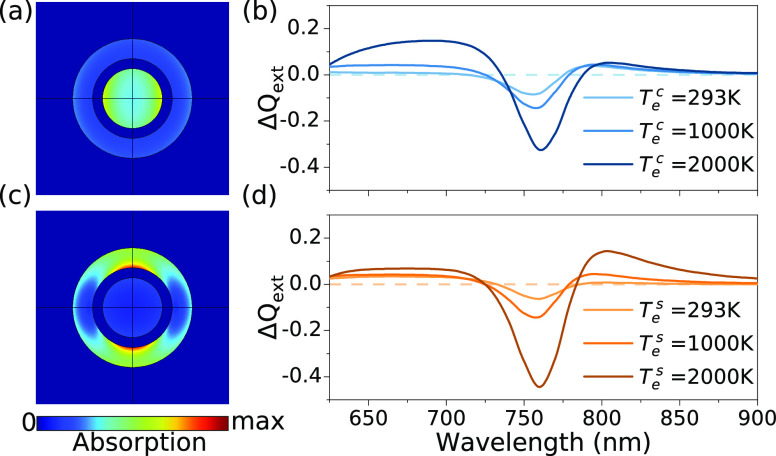
Numerical simulations of the transient optical response of the Au/SiO_2_/Au nanoparticles. (a,c) Calculated spatial distributions of the energy absorption within the multilayered nanoparticle at the excitation wavelengths of (a) 514 nm and (c) 1028 nm. (b,d) Simulated transient absorption spectra for a single nanoparticle for elevated electron temperatures in (b) the core (*T*_*e*_^*c*^) and (d) the shell (*T*_*e*_^*s*^). The nanoparticle parameters are as in Figure 2.

Simulations of the optical response of the excited nanoparticles (Figure 3b,d), based on the independent setting of the elevated electron temperature in the core (*T*_*e*_^*c*^) and/or in the shell (*T*_*e*_^*s*^), show that the increase of *T*_*e*_^*c*^ mainly modifies the short-wavelength side of the transient absorption spectrum (600–700 nm), influenced by the tail of the high-energy extinction peak, while the increase of *T*_*e*_^*s*^ primarily impacts the low-energy resonance, where the electric field is mainly localized in the gold shell (750–900 nm). The asymmetric shape of the experimental transient absorption spectra (Figure 2b,d) and their dependence on the excitation wavelength is clearly reproduced taking into account spatially nonuniform absorption in the hetero-nanoparticle. Therefore, such nanoparticles have the capability to manipulate the ultrafast nonlinear response in the spectral domain, through engineering the spatial distribution of the hot electrons. Spatial reshaping of the hot electron distribution to control the response time of the plasmonic anisotropic system was demonstrated previously with gold nanorods.^35^

Fast equilibration of the temperature between the hot carrier gas and the lattice leads to a sharp rise of the temperature of the crystal lattice, subsequently leading to lattice thermal expansion of the nanoparticle materials. This, in turn, drives the acoustic vibration modes of the nanoparticles.^19^ Such acoustic modes reveal themselves in the transient absorption spectra at longer optical delays as periodic oscillations (Figure 4a). The transient absorption, different for different pump wavelengths, shows the simultaneous presence of several periodic signals in the transient response. The spectrum of the corresponding vibrations can be analyzed with fast Fourier transform revealing two dominant oscillation frequencies (Figure 4b,c).

**Figure 4:**
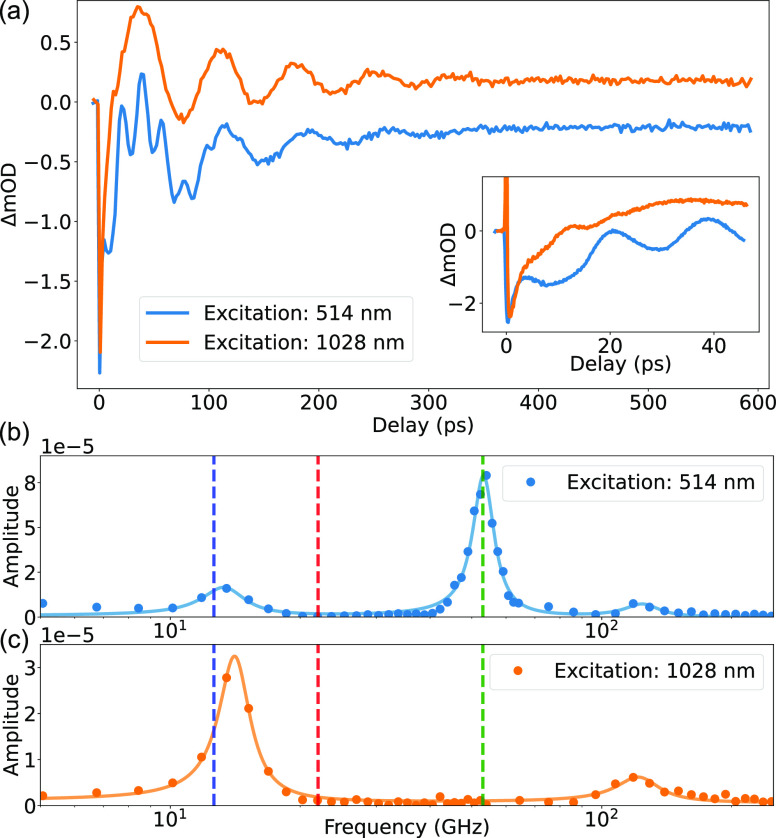
(a) Transient absorption signal measured at a wavelength of 710 nm for the two excitation conditions. The inset shows the zoom for the shorter delay range. (b,c) Fourier transforms of the transient signals in (a): (symbols) FFT, (solid lines) fit with Lorentz oscillators, and (dashed vertical lines) natural frequencies of the free acoustic vibrations of (blue) an individual gold shell, (green) an individual gold core, and (red) a multishell hetero-nanoparticle with strong mechanical coupling between a gold core and a gold shell.

In order to classify the vibrations of the multishell nanoparticles, the classical approach based on continuous mechanics to describe the free vibrations of a sphere was employed.^36^ It can be applied to arbitrary multilayer structures with spherical symmetry by setting the appropriate set of boundary conditions^37,38^ (see Supporting Information). It should be noted that while more advanced *ab initio* treatments, such as the ones based on molecular dynamics (MD) simulations, exist,^33^ it has been shown experimentally that for the nanoparticles in the range of tens of nanometers in diameter and bigger, the implemented classical approach based on the linear elasticity theory produces results that agree well with the measurement.^19^ It has been also noted that in the nanoparticles containing more than one layer, such as the core–shell nanoparticles studied here, the properties of the mechanical contact between the materials are important as they define whether the coupling between layers is strong or weak, which influences the natural frequency of the vibrations.^39^

For any nanoparticle with spherical symmetry, the possible vibrations can be divided into families of spheroidal modes (with nonzero radial displacement) and torsional modes (with zero radial displacement), and each of them is further classified by the pair of numbers (*l*, *n*) determining the angular momentum and a mode overtone, respectively.^36,40^ For the nanoparticle vibration driven by a uniform thermal expansion, only spheroidal modes with *l* = 0 are allowed by the symmetry and observed in transient optical experiments.^40^ Therefore, when the Au/SiO_2_/Au nanoparticle is excited nonresonantly at 1028 nm, most of the optical absorption and, therefore, thermal expansion happen mainly in the gold outer shell (Figure 3c) and the mode that is excited most efficiently is the fundamental (0,0) spheroidal mode of the hollow shell. The frequency of this mode is estimated to be 12.8 GHz, in agreement with the experimental observation of 14 GHz (Figure 4b,c).

When the excitation wavelength is tuned to be resonant with the short-wavelength extinction peak, optical absorption mainly takes place within the 60 nm gold core (Figure 3a), which efficiently drives the fundamental (0,0) spheroidal mode of the core. Thus, the new peak appears (Figure 4b) in the vibrational spectrum at 54 GHz (theoretical estimate is 53 GHz). The acoustic vibration of the shell is still present under 514 nm excitation, yet weaker, being indirectly excited either by weak mechanical coupling between the gold layers through the silica or by the still present yet lower optical absorption in the gold shell.

Lastly, a third heavily damped peak is visible in the spectra around 120 GHz. This frequency does not agree with the expected frequencies of the fundamental modes or their overtones for either the gold core or shell and is identified as a breathing mode of freestanding 27 nm solid gold nanoparticles (frequency estimate is 113 GHz), a small number of which are present in the solution as a byproduct of the fabrication process (see Supporting Information).

The decay time constants for all the observed acoustic vibration modes do not depend on the excitation wavelength and are of the order of 50–100 ps, several times shorter than the decay times for vibrations of gold nanoparticles in aqueous environment caused by the energy loss due to sound emission in water.^41−43^ The analytical treatment employed here can be in fact readily extended to estimate homogeneous line widths of vibrating spherical nano-objects in arbitrary viscoelastic media.^44^ Such calculations for our nanoparticles dispersed in water yield line widths of the order of 0.3 GHz, somewhat smaller than reported in the single particle experiments, due to neglect of intrinsic damping mechanisms in gold,^43^ and much smaller than observed in our experiments. The latter is probably related to inhomogeneous broadening due to the variation of thicknesses of the gold core and the shell which also affects the linear absorption spectra (Figure 1c).

It is important to note that none of the observed modes agree with the natural frequency of the fundamental vibration (22 GHz) of the nanoparticles with fully connected multilayers, which together with the separate observation of the vibrations of the gold core and the gold shell signifies that only weak mechanical coupling exists at the interface between the silica and gold shells (similar weak vibrational coupling was observed between gold shells grown on silica cores^45,46^). The mechanical contact at the interface between the gold core and the silica shell may demonstrate good mechanical coupling and lead to the deviation from the natural frequencies of the pure gold core.^40,47^ This deviation was only apparent in the optical experiments with the particles encapsulated in thick silica shells,^47^ whereas for the nanoparticles studied here, with the radii ratio  1.3, the difference in the natural frequencies of bare and encapsulated particles lies within the margins of the experimental error.

In conclusion, we have studied the fundamentals of transient optical and photoacoustic responses of the Au/SiO_2_/Au hetero-nanoparticles under various photoexcitation conditions. We observed a broad and complex transient absorption spectrum spanning the visible range, as well as a rich vibrational spectrum reported for the first time in such a plasmonic system. We have demonstrated that weak mechanical coupling between the two gold layers in the nanoparticles gives two separate acoustic vibrations of the gold core and the gold shell, that could be independently optically controlled by tuning the wavelength of the excitation. This observation not only expands the knowledge about the internal structure of composite plasmonic nanoparticles but also allows for an additional degree of freedom to control their optical and mechanical properties. These studies can be further extended to metaparticles composed of multiple shells and optical resonances, extending the vibrational spectrum and wavelength selectivity of their acousto-optical response. However, as the frequencies of subsequent shells are inversely proportional to their diameter, our experiments performed on five-layer metaparticles did not allow us to clearly observe the vibration of the second shell (at an estimated frequency of 6.7 GHz) due to strong inhomogeneous broadening, which can be overcome in the experiments on single nanoparticles.

Apart from a broadband Kerr nonlinearity important in laser physics, wavelength-selective acoustic response is important in photoacoustic imaging at different frequencies with the same nanoparticle transducer and, in reverse, can be used for sensing broadband acoustic signals. Using the weak mechanical coupling between a shell and a core, self-referenced pressure sensing can be envisaged with the pressure influencing the vibration of the shell but not the core. Selective excitation of vibrational modes of molecules coupled to hetero-nanoparticles may have a potential for controlling the energy conversion and transport in the molecules and may serve as a platform for quantum optomechanics.

## Data Availability

All the data supporting the findings of this work are presented in the text and Supporting Information and are available from the corresponding author upon reasonable request.

## Supporting Information Available

Hetero-nanoparticles fabrication; pump–probe measurements; numerical simulations; calculations of natural vibration frequencies; coherent cubic nonlinearity of water (PDF)
