# Supplementary material for: Broadband Transient Response and Wavelength-Tunable Photoacoustics in Plasmonic Hetero-nanoparticles
Source: Nano Lett. 2023 Mar 16;23(7):2786–91. doi: 10.1021/acs.nanolett.3c00063 (PMC10103169; doi:10.1021/acs.nanolett.3c00063)
Supplement: Supplementary file 1 — nl3c00063_si_001.pdf [file nl3c00063_si_001.pdf]

Supporting Information
Broadband transient response and wavelength-tuneable photoacoustics in plasmonic hetero-nanoparticles

Anton Yu. Bykov,[*,†,‡] Yuanyang Xie,[*,†,‡] Alexey V. Krasavin,[†] and Anatoly V. Zayats[†]

†*Department of Physics and London Centre for Nanotechnology, King's College London, London, WS2R 2LS, UK*

‡*These authors contributed equally to the work.*

E-mail: anton.bykov@kcl.ac.uk; yuanyang.xie@kcl.ac.uk

1. Methods.

1.1 Hetero-nanoparticles fabrication.

The studied nanoparticles were fabricated starting from commercial gold nanospheres with a nominal diameter of 60 nm (actual diameter determined experimentally from TEM images is 57.3 nm). First, a SiO_2 shell was coated onto the gold nanospheres using the modified *Stöber* method.[1] After SiO_2 /Au was functionalised with Au seeds, an Au shell was grown by the reduction of $HAuCl_4$ with formaldehyde (CH_2O) in a plating solution. Based on the dynamic light scattering measurements, a polydispersity index (PDI) of 0.12 indicate that the nanoparticle are close to monodisperse.

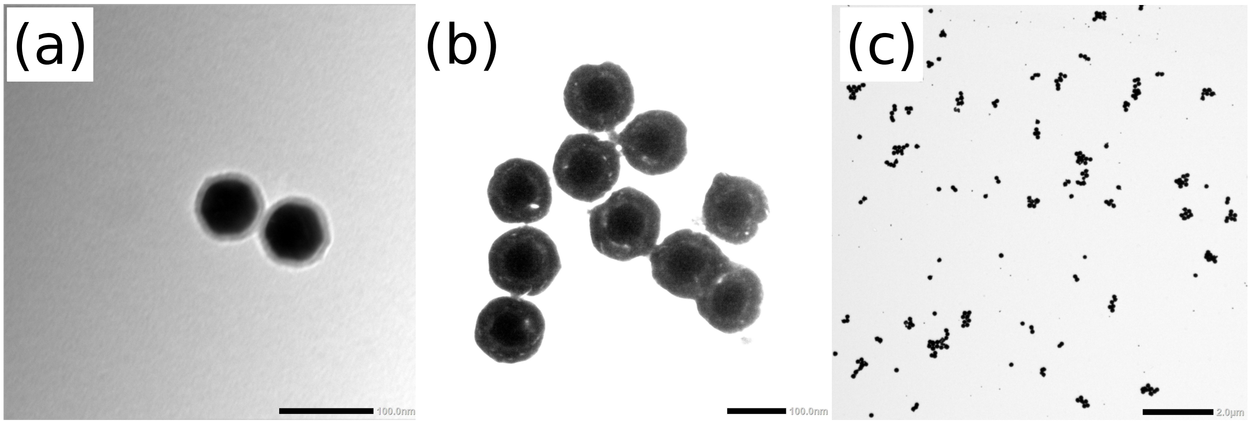

Figure S1: TEM images of (a) Au/SiO_2 nanoparticles and (b,c) Au/SiO_2/Au hetero-nanoparticles under different magnifications (please note that the nanoparticles tend to aggregate when deposited on a TEM substrate). The presence of the outer gold shell greatly reduces the visibility of the multilayer structure in the TEM images. In (c), the dominant nanoparticles are Au/SiO_2/Au while smaller dot-like features are single ∼27 nm Au nanoparticles present in the solution as byproduct of the synthesis. The scale bar is 100 nm in (a, b) and 2 μm in (c).

SiO_2 shell coating. Before being coated by SiO_2, the Au nanoparticles (Au60) were functionalized by mixing with a SH-PEG solution for at least 5 hours. Then, 16 mL of an Au60 solution (1 OD) was added to 6.4 mL of a functionalized-Au60 ethanol solution. The growth of SiO_2 was achieved using the classic Stöber method.[1] Briefly, 1.6 mL of a TEOS solution (2 μL in 2 mL ethanol) and 48 μL of $NH_3 \cdot H_2O$ were added to the functionalized-Au60 ethanol solution. Then, the mixture was further stirred overnight. After being washed with ethanol three times, the SiO_2 coated particles (SiO_2/Au) were dispersed in 14.8 mL of ethanol and were stored at 4 °C.

Gold shell growing. The Au seeds (2 nm diameter) were prepared using the method demonstrated by Duff et al.,[2] the plating solution was prepared by mixing 50 mL of water, 12.5 mg of K_2CO_3, and 0.75 mL of an 1 wt% aqueous $HAuCl_4$ solution, and was aged at 4 °C overnight. Then, excessive APTMS was used to modify 6 mL SiO_2/Au nanoparticles to provide amine groups for the attachment of the Au seeds on their surfaces. The solution was centrifuged to remove unattached Au seeds and dispersed in 1 mL of DI water. The Au-seed-SiO_2/Au solution was mixed with a 8 mL plating solution as a source of Au^{3+} and

34 μL of CH_2O as a reductant. The gold shell appeared after 15 min further stirring. The thickness of the Au shell can be controlled by changing the ratio of Au-seed-SiO_2/Au and CH_2O.

Chemicals. Tetraethyl orthosilicate (TEOS, 98%), ammonium hydroxide solution ($NH_3 \cdot H_2O$, 28%), absolute ethanol, (3-Aminopropyl)trimethoxysilane (APTMS, 97%), Au(III) chloridetrihydrate ($HAuCl_4 \cdot {}_3H_2O$, 99.9%), tetrakis(hydroxymethyl)phosphonium chloride (THPC, 80% in water), Au(III) chloridetrihydrate ($HAuCl_4 \cdot {}_3H_2O$, 99.9%), potassium carbonate (K_2CO_3, 99.995%), O-[2-(3-mercaptopropionylamino)ethyl]-methylpolyethylene glycol (SH-PEG), formaldehyde solution (CH_2O, 37 wt%), sodium hydroxide (NaOH, 98%), sodium chloride (NaCl, 99%) and hydrochloric acid (HCl, 36%) were purchased from Sigma-Aldrich. Gold nanospheres 60 nm in diameter (Au60) were received from BBI Solutions. The water with a resistivity of 18.2 $M\Omega \cdot cm^{-1}$ (DI water) was used throughout the procedures.

1.2 Pump-probe measurements.

The transient optical measurements were performed in a collinear configuration with an 1028 nm output (or 514 nm SHG) of an Yb:KGW amplifier (Light Conversion Pharos, 230 fs pulses at 200 kHz) as a control beam and a tuneable output of an OPA (Light Conversion Orpheus, 150 fs) as a probe beam. The path length difference between the pump and probe beams is adjusted using a mechanical delay line. The probe beam is divided into a signal and reference beams, both focused non-collinearly into a cuvette with the nanoparticles with a 25x reflective objective (Thorlabs LMM-25x-UVV). The signal and pump beams were made collinear by a mirror arrangement so that only the propagation of the signal beam, but not the reference, is influenced by the pump beam. The signal and the reference were then aligned on the two photodiodes of a balanced detector. The detector was monitored with a lock-in amplifier (Stanford Research SR865A), which uses as its reference the frequency of an optical chopper positioned in the pump path. The probe wavelength can be tuned in the range 600–900 nm, which corresponds to the spectrum of interest for the plasmonic

nanoparticles studied here. Simultaneous propagation of the signal and reference beams through the cuvette leads to automatic self-referencing when tuning the wavelength, allowing to record transient absorption spectra.

1.3 Numerical simulations.

Scattering on spherical Au/SiO_2/Au multishells in water was simulated using the finite element method (COMSOL Multiphysics software) in a scattered field formulation. A single nanoparticle with the experimental geometric parameters (60 nm gold core, 10 nm silica shell, 20 nm gold shell) was simulated. The simulation domain was surrounded by a perfectly matched layer to assure the absence of back-reflection. The extinction efficiency was calculated dividing an extinction cross-section σ_{ext} by its geometrical counterpart σ_{geom}. The extinction cross-section σ_{ext} is a sum of scattering (σ_{scat}) and absorption (σ_{abs}) cross-sections, which were obtained by integrating the scattered field power flow over a surface surrounding the nanoparticles and the total field power flow entering them, respectively, followed by the normalisation by the incident power flow. The energy absorption maps present the power dissipation density.

The optical properties of gold at various electron temperatures T_e were calculated using the following approach. The gold permittivity can be decomposed into the contribution defined by interband transitions between occupied d-band states and unoccupied states in a hybridised sp-band ($\epsilon_{inter}(\omega, T_e)$), and the intraband contribution of the conduction electrons in the sp-band ($\epsilon_{intra}(\omega, T_e)$):

$$\epsilon_{Au}(\omega, T_e) = \epsilon_{inter}(\omega, T_e) + \epsilon_{intra}(\omega, T_e). \tag{S1}$$

In the visible spectral range, the interband permittivity of gold is defined by three optical transitions, one near the X point, and two near the L point (L_4^+ and L_{5+6}^+).[3] The imaginary part of the permittivity related to these transitions can be calculated using a theoretical

approach developed by Rosei et al.:[4,5]

$$Im\left[\epsilon_{inter}(\omega, T_e)\right] = \frac{A_X J_X(\omega, T_e) + A_{L_4^+} J_{L_4^+}(\omega, T_e) + A_{L_{5+6}^+} J_{L_{5+6}^+}(\omega, T_e)}{\hbar\omega^2}, \tag{S2}$$

where

$$J_j(\omega, T_e) = \int_{E_{min}^j}^{E_{max}^j} \int D_j(\omega', E)(f_l(E - \hbar\omega', T_e) - f_u(E, T_e))B(\omega' - \omega, E, T_e)\, d\omega' dE \tag{S3}$$

is the joint density of states (JDOS) for the corresponding transitions with $j = X, L_4^+, L_{5+6}^+$. It is obtained by integration of the JDOS energy distribution $D_j(\omega', E)$ over the excited states with an energy E in the conduction band taking into account Fermi-Dirac distributions $f_u(E, T_e)$ and $f_l(E - \hbar\omega', T_e)$ of the carriers in the lower (l) and upper (u) bands, respectively, and energy- and temperature-dependent broadening of the upper (sp-band) due to electron scattering:

$$B(\omega' - \omega, E, T_e) = \frac{1.76}{2b\gamma(E, T_e)} \cosh^{-2}\left(\frac{1.76\,(\omega' - \omega)}{b\gamma(E, T_e)}\right), \tag{S4}$$

which is similar to Lorenzian, but with exponential tails presenting a beter approximation of the transition lineshape in the considered wavelength range.[3,6] The total electron scattering rate

$$\gamma(E, T_e) = \gamma_{el-ph} + \gamma_{el-el}(E, T_e) \tag{S5}$$

has contributions from electron-phonon scattering γ_{el-ph} and energy-dependent electron-electron scattering

$$\gamma_{el-el}(E, T_e) = \frac{\pi^3 \Gamma \Delta}{12\hbar E_F}\left((k_B T_e)^2 + (E/2\pi)^2\right), \tag{S6}$$

where $E_F = 5.51$ eV is the Fermi energy, $\Gamma = 0.55$ is the scattering probability averaged over the Fermi surface and $\Delta = 0.77$ is the fractional umklapp scattering.[7,8] The interband transmission coefficients $A_X/A_{L_4^+} = 1$ and $A_X/A_{L_{5+6}^+} = 0.4$ related to the values of the

corresponding transition dipole moments, and broadening coefficient $b = 2.5$ were set to fit the Johnson-Cristy data for the permittivity of gold at room temperature.[3,6,9,10] The real part of the interband permittivity $Re\left[\epsilon_{inter}(\omega, T_e)\right]$ was found from its imaginary part $Im\left[\epsilon_{inter}(\omega, T_e)\right]$ using the Kramers–Kronig relations and an approach based on the Hilbert transform described in Ref.[11]

The intraband component of the permittivity is given by the Drude formula

$$\epsilon_{intra}(\omega, T_e) = \epsilon_{inf} - \frac{\omega_p^2}{\omega^2 + i\omega\gamma(\hbar\omega, T_e)}, \tag{S7}$$

where $\epsilon_{inf} = 3$ was set to fit the Johnson-Cristy data for the permittivity of gold at room temperature[?] and $\omega_p = 9.02$ eV is the gold plasma frequency.

2. Calculations of natural vibration frequencies.

Free vibrations in vacuum of a core, a shell and a complete multishell nanoparticle can be easily obtained following Erdinger and Suhubi.[12] For spheroidal vibrations at frequency Ω, with l = 0, that are of interest in this work, in the absence of dissipation, the displacements and tractions can be expressed in spherical coordinates as

$$\begin{aligned}
u_r(r) &= Aqz_1(qr)e^{-i\Omega t}, \\
u_\theta(r) &= Az_0(qr)e^{-i\Omega t}, \\
t_{rr}(r) &= A\frac{2\mu_L}{r}\left(-\frac{Q^2r^2}{2}z_0(qr) + 2qrz_1(qr)\right)e^{-i\Omega t}, \\
t_{r\theta}(r) &= -A\frac{2\mu_L}{r}\left(z_0(qr) - qrz_1(qr)\right)e^{-i\Omega t},
\end{aligned} \tag{S8}$$

where C_L and C_T are the longitudinal and transverse speeds of sound in the material, respectively, $q = \Omega/C_L$, $Q = \Omega/C_T$, μ_L is the Lame parameter of material, A is the vibration mode amplitude, and $z_{0,1}$ are the linear combinations of spherical Bessel functions $j_{0,1}$ and $y_{0,1}$ in all regions except for the one that contains the origin, where only $j_{0,1}$ are used. Note

that we omitted here u_ϕ and $t_{r\phi}$ as these components are related to torsional modes only, and are not of interest in this work as they cannot be optically excited. The free vibrations can be obtained by setting the appropriate boundary conditions for the displacement and traction: continuity on internal boundaries and the absence of traction on free boundaries. This method produces a system of 2, 4, and 10 linear equations for the cases of a sphere, a spherical shell and a hetero-particle, respectively. The frequencies of the free vibrations can be obtained by setting the determinant of the system matrix to zero and obtaining the roots numerically. The frequencies quoted in the main text are obtained with this method, using a custom numerical solver written in Python.

Separately, we used an extended version of this approach as proposed in Ref.[13] to evaluate the damping of mechanical vibrations due to the presence of water. The method consists of searching for the solution in the surrounding medium as decaying spherical waves propagating at speeds

$$\begin{aligned} C_L &= \sqrt{\frac{\lambda_L + i\Omega\lambda + 2(\mu_L + i\Omega\mu)}{\rho}}, \\ C_T &= \sqrt{\frac{\mu_L + i\Omega\mu}{\rho}}, \end{aligned} \tag{S9}$$

where λ and μ are the viscosity coefficients of the surrounding medium, and λ_L, μ_L are its Lame parameters, related to the transversal/longitudinal speeds of sound, and the Bessel functions for the displacement given by a set of equations S8, are replaced with the Hankel functions of the second kind in order to fulfil the correct asymptotic behaviour at infinity. The calculation may then proceed in a similar manner to the one mentioned above, with the exception that solutions are now searched with complex frequencies.

For the considered geometries, we have evaluated the damping and the shift of the real part of the frequency for the hetero-nanoparticle in water, as well as a gold spherical shell either having water both on the inside and outside or possessing a free surface on the inside and surrounded by water on the outside. In all cases, the damping was shown to have a

minor effect (within few percent) on the real part of the normal frequency, which justified the use of the model without damping, the linewidths around 0.3 GHz were obtained from the imaginary part of the normal frequencies in all cases.

3. Coherent cubic nonlinearity in water

Figure S2 shows the transient absorption spectra recorded under the 1028 nm excitation in a cuvette filled with pure deionised water, obtained under similar conditions as in Fig. 2. While the long-lived induced transient absorption/bleaching signal, indicative of hot carrier dynamics in gold, is absent in pure water, the short-lived positive signal is still present. Assuming that this coherent nonlinearity arises from efficient pumping into a vibrational level of water molecules with the difference frequency $\Omega = \omega_{probe} - \omega_{pump}$, a good agreement with the known spectral features of the absorption spectra of water is obtained.[14,15]

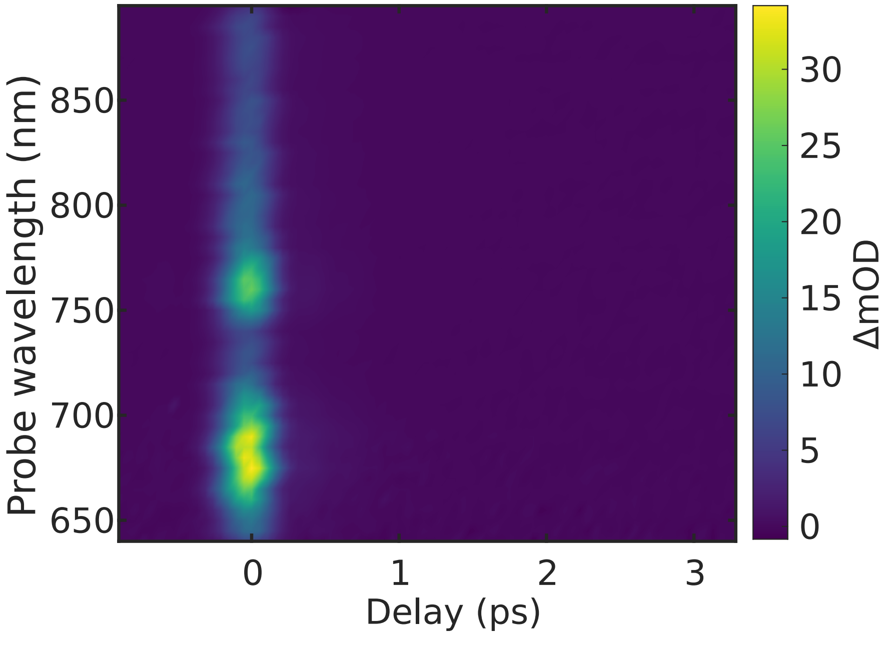

Figure S2: Transient absorption spectra recorded in a cuvette filled with pure deionised water under 1028 nm excitation.

References

(1) Shinoda, K.; Saito, H. The effect of temperature on the phase equilibria and the types of dispersions of the ternary system composed of water, cyclohexane, and nonionic surfactant. *Journal of Colloid and Interface Science* **1968**, *26*, 70–74.

(2) Duff, D. G.; Baiker, A.; Edwards, P. P. A new hydrosol of gold clusters. 1. Formation and particle size variation. *Langmuir* **1993**, *9*, 2301–2309.

(3) Masia, F.; Langbein, W.; Borri, P. Measurement of the dynamics of plasmons inside individual gold nanoparticles using a femtosecond phase-resolved microscope. *Phys. Rev. B* **2012**, *85*, 235403.

(4) Rosei, R. Temperature modulation of the optical transitions involving the Fermi surface in Ag: Theory. *Phys. Rev. B* **1974**, *10*, 474–483.

(5) Guerrisi, M.; Rosei, R.; Winsemius, P. Splitting of the interband absorption edge in Au. *Phys. Rev. B* **1975**, *12*, 557–563.

(6) Zilli, A.; Langbein, W.; Borri, P. Quantitative measurement of the optical cross sections of single nano-objects by correlative transmission and scattering microspectroscopy. *ACS Photonics* **2019**, *6*, 2149–2160.

(7) Beach, R. T.; Christy, R. W. Electron-electron scattering in the intraband optical conductivity of Cu, Ag, and Au. *Phys. Rev. B* **1977**, *16*, 5277–5284.

(8) Lawrence, W. E. Electron-electron scattering in the low-temperature resistivity of the noble metals. *Phys. Rev. B* **1976**, *13*, 5316–5319.

(9) Johnson, P. B.; Christy, R. W. Optical constants of the noble metals. *Phys. Rev. B* **1972**, *6*, 4370–4379.

(10) Heilpern, T.; Manjare, M.; Govorov, A. O.; Wiederrecht, G. P.; Gray, S. K.; Harutyunyan, H. Determination of hot carrier energy distributions from inversion of ultrafast pump-probe reflectivity measurements. *Nat. Comm.* **2018**, *9*, 1853.

(11) Castro, F.; Nabet, B. Numerical computation of the complex dielectric permittivity using Hilbert transform and FFT techniques. *J. Franklin Inst.* **1999**, *336B*, 53–64.

(12) Eringen, A.; Suhubi, E. S. *Elastodynamics*; Academic Press, New York, 1975; pp 717–862.

(13) Saviot, L.; Netting, C. H.; Murray, D. B. Damping by bulk and shear viscosity of confined acoustic phonons for nanostructures in aqueous solution. *The Journal of Physical Chemistry B* **2007**, *111*, 7457–7461.

(14) Ockman, N. The infra-red and Raman spectra of ice. *Advances in Physics* **1958**, *7*, 199–220.

(15) Hale, G. M.; Querry, M. R. Optical constants of water in the 200 nm to 200 μm wavelength region. *Appl. Opt.* **1973**, *12*, 555–563.
